# A Medical Fellowship

**Published:** 1919-01-25

**Authors:** 


### A MEDICAL FELLOWSHIP.

It is a natural and happy inspiration which suggests that advantage should be taken of existing circumstances to accentuate the sense of comradeship among the medical practitioners of the various countries which have been engaged in the war. Medical sympathies have never been restricted by differences of race and language, and the common task has always been a fairly effective bond. The interests of the craft are common to all countries, and the sense of a shared responsibility has largely banished particularism. This basis, of course, still exists, and though exclusion in certain directions is almost inevitable, work achieved, whatever be its source, must needs be a common possession. But recent events and present opportunities offer something more than this. There has been a sharing of special enterprises and dangerous emergencies. Medicine in all the Allied countries has had to meet warlike developments which, had they not been successfully encountered, might have been fatal to military success, and the duty has meant a united effort. Thus among medical practitioners has grown up not only a union inspired by patriotism and the bond of a common cause, but also a sense of a shared special contribution by the profession to which they belong. In the very nature of things this is specially true of English-speaking practitioners brought from the various ends of the earth and personally associated by the pressure of great events. The centre for these is obviously this country, and it has been a duty of hospitality as well as a manifestation of good will that our medical organisations should offer to our *confrères* from over the seas whatever opportunity lay within their power. That this has been appreciated is certain, and the results cannot be other than pleasing. It is now felt that something more manifest and more permanent should be attempted. The suggestion is to establish a Fellowship of Medicine, which shall extend beyond the English-speaking peoples to all the countries which have been associated in the struggle and victory of the Great War. It is hoped, not unreasonably, that this will be a good and pleasant thing for the medical profession, and that it will help in some measure to the establishment of mutual understanding between the nations which have suffered in the common cause. Medicine, of course, has its influence in international affairs, even if it is only by the effect its disciples exercise on their immediate circles, and knowledge and sympathy are perhaps as necessary as treaties and protocols. Every one, therefore, may wish success to the proposed Fellowship of Medicine. The difficulty at first sight is to find an organ and opportunity of expression. Interchange of lectures and visits from distinguished physicians and surgeons have their value and we hope to see them continued and multiplied, and ceremonial occasions as chance offers are by no means to be despised. But all these are somewhat remote from the general body of the profession. In our judgment one of the most effective contributions to the desired end would be an interchange of students between the Universities of the several countries concerned, and if any Fund is to be raised for the purposes of the Fellowship it is in this direction we should be inclined to expend it. The impressionable age is the time for sowing the seeds of sympathy and understanding, and it is under these conditions that a full harvest may be expected. Certainly any enterprise which boasts Sir William Osler as President and Mr. J. Y. W. MacAlister as Secretary starts its career under favourable auspices.

